# Right-Lateralized Enhancement of the Auditory Cortical Network During Imagined Music Performance

**DOI:** 10.3389/fnins.2022.739858

**Published:** 2022-02-10

**Authors:** Shoji Tanaka, Eiji Kirino

**Affiliations:** ^1^Department of Information and Communication Sciences, Sophia University, Tokyo, Japan; ^2^Department of Psychiatry, Juntendo University School of Medicine, Tokyo, Japan; ^3^Juntendo University Shizuoka Hospital, Shizuoka, Japan

**Keywords:** asymmetry, fMRI, functional connectivity, laterality, imagery, network

## Abstract

Although the primary role of the auditory cortical areas is to process actual sounds, these areas are also activated by tasks that process imagined music, suggesting that the auditory cortical areas are involved in the processes underlying musical imagery. However, the mechanism by which these areas are involved in such processes is unknown. To elucidate this feature of the auditory cortical areas, we analyzed their functional networks during imagined music performance in comparison with those in the resting condition. While imagined music performance does not produce any musical sounds, the participants heard the same actual sounds from the MRI equipment in both experimental conditions. Therefore, if the functional connectivity between these conditions differs significantly, one can infer that the auditory cortical areas are actively involved in imagined music performance. Our functional connectivity analysis revealed a significant enhancement in the auditory network during imagined music performance relative to the resting condition. The reconfiguration profile of the auditory network showed a clear right-lateralized increase in the connectivity of the auditory cortical areas with brain regions associated with cognitive, memory, and emotional information processing. On the basis of these results, we hypothesize that auditory cortical areas and their networks are actively involved in imagined music performance through the integration of auditory imagery into mental imagery associated with music performance.

## Introduction

Cortical auditory processing is essential for both performing and listening to music. Similar to visual processing (Felleman and Van Essen, 1991), cortical auditory signal processing has been suggested to be distributed in a hierarchical manner (Rauschecker and Scott, 2009; Okada et al., 2010; Peelle et al., 2010; Hackett, 2015). The primary auditory cortex is located in Heschl’s gyrus (HG) (Brewer and Barton, 2016), which mediates fundamental frequency analysis of complex sounds (Hall and Plack, 2009; Wang, 2018). The auditory cortex extends posteriorly to the planum temporale (PT) and anteriorly to the planum polare (PP) (Peelle et al., 2010; Hackett, 2015), where sounds are further analyzed. Sounds with pitch activate HG more than those without pitch, whereas sounds that vary in pitch to produce a melody activate HG, PT, and PP. Griffiths and Warren (2002) proposed that the PT is a computational hub of spectrotemporal information, which is gated to higher-order cortical areas for further processing, allowing object recognition, and auditory space perception (Griffiths and Warren, 2002). The PP has been suggested to play an important role in the perceptual integration of simpler and shorter sound fragments to form more complex patterns, such as tones to melodies and words to sentences (Baumann et al., 2007). Moreover, in comparison with other complex sounds, musical stimuli preferentially activate the PP (Angulo-Perkins et al., 2014). These findings indicate a hierarchy in musical sound processing in the way the brain processes pitch, with the center of activity moving anterolaterally away from the primary auditory cortex as the processing of melodic sounds proceeds (Patterson et al., 2002).

While the auditory cortical areas in both hemispheres are involved in the processing of sounds, hemispheric asymmetry reflects the specificities of acoustic features, and processing demands. Leftward lateralization is typically observed for temporal processing in speech, whereas rightward lateralization is observed for spectral processing in music (Warrier et al., 2009; Zatorre, 2012). However, laterality in music processing has been investigated mostly through passive tasks such as listening to a piece of music, and laterality during music performance is unknown. To date, studies on auditory processing during music performance are scarce. Music performance requires active auditory processing that is integrated with information relevant to the performance. Such integration of information would require inter-regional interaction between the auditory cortex and other regions. However, it is unclear how the auditory cortical areas cooperate with other brain regions during music performance.

In music performance, mental imagery representing the music plays a leading role. Although the primary role of the auditory cortical areas is to process actual sounds, these areas are also activated by tasks that process imagined music (Zatorre and Halpern, 2005), suggesting that the auditory cortical areas are involved in the processes underlying musical imagery. Since imagining music does not produce any sounds, exploring the roles of the auditory cortical areas in the processing of imagined music is intriguing. Previous studies have reported that the auditory cortical areas are activated during silent music reading (Hoppe et al., 2014) and timbre imagery (Halpern et al., 2004), as well as an inter-subject correlation of auditory cortical activity during melodic imagery (Regev et al., 2021), suggesting that the auditory areas could play a role in imagined music performance. A recent study suggested that imagined singing of an aria constructed an “embodied scene” in the precuneus, the center for mental imagery processing, and its networks (Tanaka and Kirino, 2021). Musical imagery studies have reported rightward asymmetry in activity (Halpern et al., 2004; Regev et al., 2021) and connectivity between the precuneus and the auditory cortical areas (Tanaka and Kirino, 2021). To elucidate how the auditory cortical areas are involved in the processing of musical imagery, we analyzed the reconfiguration of their functional networks during imagined music performance. The results of this analysis can indicate how auditory areas contribute to imagined music performance. In this study, functional connectivity was estimated using functional magnetic resonance imaging (fMRI) data from imagined music performances and resting conditions. We extracted connections whose connectivities with the auditory cortical areas differed significantly from that in the resting condition.

## Materials and Methods

### Ethical Issues

All study procedures were approved by the ethics committees of Sophia University and Juntendo University, Japan. This study conformed to the tenets of the Declaration of Helsinki. All participants provided written informed consent before participating in the study.

### Participants

We recruited 41 graduate and undergraduate music school students (mean age, 23.4 years; age range, 19–30 years). All participants were healthy, right-handed Japanese women, with no history of neurological or neuropsychiatric disease. The students majoring in music had begun musical training at the age of 3–5 years (i.e., all participants had more than 15 years of musical training) and had actively participated in concert performances. These students specialize in classical music playing on various instruments: 15 play the piano, 8 play the violin, 4 play the clarinet, and 14 are vocalists.

### Task

All participants underwent two fMRI sessions: an imagined music performance session followed by a resting-state session. Each session lasted 6 min and 40 s. During the imagined music performance session, the participants were asked to imagine the act of music performance in a concert hall as vividly as possible without performing actual movements with their eyes closed. The music performed was chosen from their repertoires. For example, pianists chose a piece of piano music (e.g., Ballade No. 1 by Frederic Chopin), violinists chose a piece of violin music (e.g., Violin Sonata No. 1 by Robert Schumann), and vocalists chose an opera aria (e.g., “Regnava nel silenzio” from Lucia di Lammermoor by Donizetti). The performance was truncated at the end of each session. In the resting-state session, the participants were instructed not to think of anything in particular with their eyes closed.

### Image Acquisition

Whole-brain images were acquired using a Philips Achieva 3.0-T MRI scanner equipped with a 32-channel head coil array. We collected high-resolution T1-weighted images for anatomical reference, using a 3D magnetization-prepared rapid acquisition gradient echo sequence with the following parameters: echo time (TE) = 3.3 ms, repetition time (TR) = 15 ms, flip angle = 10°, matrix size = 180 × 256 × 256, and voxel size = 1 mm × 1 mm × 1 mm. The total image acquisition time was 3 min and 31 s.

We collected blood oxygenation level-dependent (BOLD) fMRI data during the imagined music performance and resting-state sessions. A T2*-weighted gradient-echo-planar imaging sequence was used with the following parameters: TE = 30 ms, TR = 2000 ms, flip angle = 90°, field of view = 240 mm × 240 mm, matrix size = 64 × 64, number of axial slices = 33, and voxel size = 3.75 mm × 3.75 mm × 4.00 mm. The slices were acquired in the interleaved ascending order, starting with odd-numbered slices followed by even-numbered slices. Each session consisted of 200 scans. The image acquisition time was 6 min and 40 s.

### Preprocessing

The imaging data were preprocessed using the CONN toolbox version 20.b (Whitfield-Gabrieli and Nieto-Castanon, 2012), in conjunction with Statistical Parametric Mapping version 12 (Wellcome Department of Cognitive Neurology, London, United Kingdom)^1^, running on MATLAB version R2021a (MathWorks, Inc.). Individual fMRI data were co-registered with the T1-weighted images. The fMRI data were realigned, slice-timing corrected, and normalized to the standard Montreal Neurological Institute template, as implemented in the Statistical Parametric Mapping software platform. We processed image artifacts originating from head movement by using the ART-based scrubbing procedure as an artifact removal tool (Nieto-Castanon, 2020). Signal contributions from the white matter, cerebrospinal fluid, and micro-head movements (six parameters) were regressed out of the data. Finally, the fMRI data were band-pass filtered (0.008–0.09 Hz) and functional images were spatially smoothed using a Gaussian filter kernel (full width at half-maximum = 8 mm) for subsequent seed-to-voxel analysis.

### Analysis

We performed a region of interest (ROI)-to-ROI analysis of functional connectivity using the CONN toolbox. The ROI set implemented in the CONN toolbox was based on the Harvard-Oxford atlas. For each participant, we extracted the residual BOLD time courses from 132 ROIs covering the whole brain. Further, the correlation coefficients were calculated from the time courses. The correlation coefficients were converted into normally distributed scores using Fisher’s transformation. Within the same sample, we statistically tested between-condition differences in functional connectivity using a two-tailed *t*-test. The threshold for between-condition differences in the connectivity matrix was set at *p* < 0.05, with false discovery rate (FDR) correction.

## Results

**FIGURE 1:**
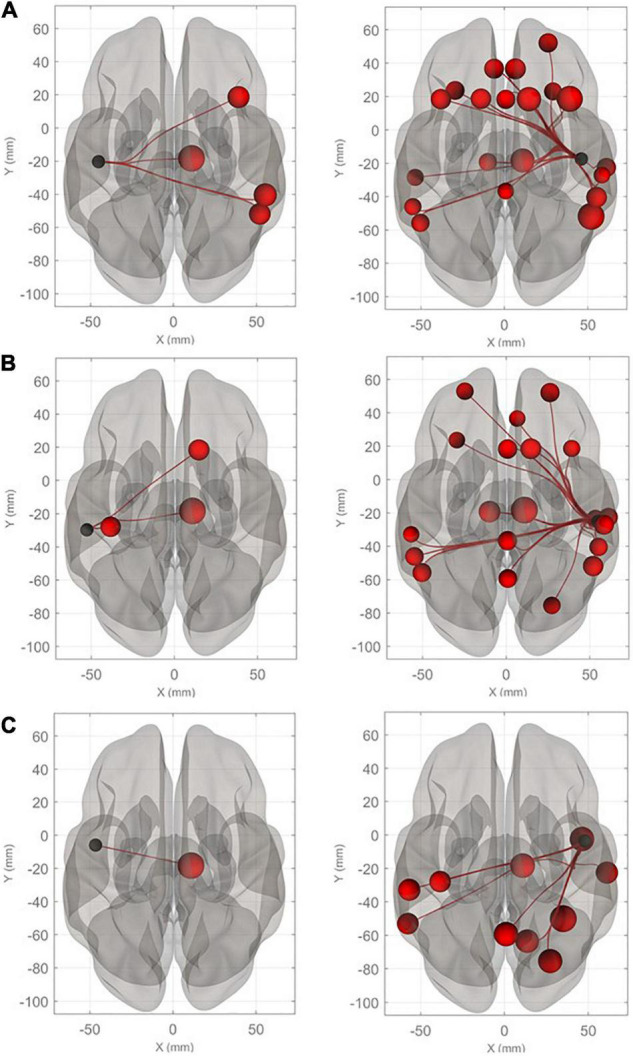
Connectivity diagrams of Heschl’s gyrus (HG), planum temporale (PT), and planum polare (PP). The seed ROIs, HG **(A)**, PT **(B)**, and PP **(C)**, are indicated by black dots. The target regions that had significantly higher connectivity with the seed ROIs (*p*-FDR < 0.005) during imagined music performance in comparison with the resting state are indicated by red spheres. The radius of the spheres varies with *T* values from the maximum (*T* = 6.22) to the minimum (*T* = 3.66). The names of the target regions are listed in Table 1.

Connectivity diagrams of HG, PT, and PP are shown in Figure 1. The seed ROIs, HG **(A)**, PT **(B)**, and PP **(C)**, are indicated by black dots. The target regions that showed significantly increased connectivity with the seed ROIs (*p*-FDR < 0.005) during the imagined performance in comparison with the resting state are indicated by red spheres. The names of the target R regions are listed in Table 1. Changes in connectivity were all increases. The diagrams clearly show the rightward lateralization of the networks with significantly higher connectivity with the seed ROIs. The right HG showed especially higher connectivity with the angular gyrus (AG), middle frontal gyrus (MFG), and superior frontal gyrus (SFG) in the right hemisphere [*T*(40) > 5.0]. A similar, but slightly less marked, pattern was extracted for the right PT. The right PP showed enhanced connectivity with the temporal, parietal, and occipital regions. All seed ROIs showed significantly higher connectivity with the thalamus.

**TABLE 1:** Brain regions that showed significant differences in functional connectivity with the auditory areas between the task and resting conditions.

ROI	Target region	Hemisphere	MNI coordinates (x, y, z) (mm)	*T*(40)	*p*-FDR
HG.L	**Thalamus**	**R**	(11, −18, 7)	**5.33**	**0.0005**
	pSMG	R	(55, −40, 34)	4.64	0.0024
	MFG	R	(39, 19, 43)	4.42	0.0031
	AG	R	(52, −52, 32)	4.34	0.0031
HG.R	**AG**	**R**	(52, −52, 32)	**6.22**	**0.0000**
	**MFG**	**R**	(39, 19, 43)	**6.03**	**0.0000**
	**SFG**	**R**	(15, 18, 57)	**5.66**	**0.0001**
	**Thalamus**	**R**	(11, −18, 7)	**5.60**	**0.0001**
	PaCG	R	(7, 37, 23)	4.84	0.0005
	pSMG	R	(55, −40, 34)	4.79	0.0005
	SFG	L	(−14, 19, 56)	4.74	0.0005
	MFG	L	(−38, 18, 42)	4.72	0.0005
	PaCG	L	(−6, 37, 21)	4.61	0.0006
	FP	R	(26, 52, 8)	4.43	0.0009
	pMTG	R	(58, −49, 2)	4.41	0.0009
	ACC		(1, 18, 24)	4.39	0.0009
	OFC	L	(−30, 24, −17)	4.32	0.0010
	AG	L	(−50, −56, 30)	4.19	0.0014
	Thalamus	L	(−10, −19, 6)	4.14	0.0015
	OFC	R	(29, 23, −16)	4.11	0.0016
	pSMG	L	(−55, −46, 33)	3.93	0.0026
	pITG	L	(−53, −28, −26)	3.90	0.0026
	PCC		(1, −37, 30)	3.76	0.0037
	aSMG	R	(58, −27, 38)	3.75	0.0037
PT.L	**Thalamus**	**R**	(11, −18, 7)	**5.59**	**0.0002**
	SFG	R	(15, 18, 57)	4.43	0.0047
	PostCG	L	(−38, −28, 52)	4.29	0.0049
PT.R	**Thalamus**	**R**	(11, −18, 7)	**6.14**	**0.0000**
	Thalamus	L	(−10, −19, 6)	4.98	0.0008
	SFG	R	(15, 18, 57)	4.73	0.0012
	AG	R	(52, −52, 32)	4.65	0.0012
	aSMG	R	(58, −27, 38)	4.52	0.0014
	ACC		(1, 18, 24)	4.40	0.0014
	PCC		(1, −37, 30)	4.37	0.0014
	FP	R	(26, 52, 8)	4.37	0.0014
	AG	L	(−50, −56, 30)	4.30	0.0015
	pMTG	R	(58, −49, 2)	4.25	0.0015
	Precuneus		(1, −59, 38)	4.23	0.0015
	pSMG	L	(−55, −46, 33)	4.21	0.0015
	pSMG	R	(55, −40, 34)	4.17	0.0016
	pITG	R	(53, −23, −28)	4.08	0.0020
	OFusG	R	(27, −75, −12)	3.94	0.0026
	FP	L	(−25, 53, 8)	3.94	0.0026
	MFG	R	(39, 19, 43)	3.87	0.0030
	OFC	L	(−30, 24, −17)	3.71	0.0044
	PaCG	R	(7, 37, 23)	3.70	0.0044
	aSMG	L	(−57, −33, 37)	3.66	0.0048
PP.L	Thalamus	R	(11, −18, 7)	4.86	0.0024
PP.R	TOFusC	R	(35, −50, −17)	4.94	0.0017
	Thalamus	R	(11, −18, 7)	4.56	0.0017
	aITG	R	(46, −2, −41)	4.51	0.0017
	Precuneus		(1, −59, 38)	4.51	0.0017
	OFusG	R	(27, −75, −12)	4.47	0.0017
	LG	R	(14, −63, −5)	4.36	0.0019
	pMTG	R	(58, −49, 2)	4.12	0.0033
	aSMG	L	(−57, −33, 37)	4.09	0.0033
	toMTG	L	(−58, −53, 1)	4.00	0.0037
	PostCG	L	(−38, −28, 52)	3.98	0.0037

*ROI, region of interest; MNI, Montreal Neurological Institute; FDR, false discovery rate; HG, Heschl’s gyrus; PT, planum temporale; PP, planum polare; ACC, anterior cingulate cortex; AG, angular gyrus; aITG, anterior inferior temporal gyrus; aSMG, anterior supramarginal gyrus; FP, frontal pole; LG, lingual gyrus; MFG, middle frontal gyrus; OFC, orbitofrontal cortex; OFusG, occipital fusiform gyrus; PaCG, paracingulate gyrus; PCC, posterior cingulate cortex; pITG, posterior inferior temporal gyrus; pMTG, posterior middle temporal gyrus; PostCG, postcentral gyrus; pSMG, posterior supramarginal gyrus; SFG, superior frontal gyrus; TOFusC, temporal occipital fusiform cortex.*

*Bold represents regions that showed marked increase in connectivity [T(40) > 5.0].*

## Discussion

The present study compared the functional connectivity of the auditory cortical areas (HG, PT, and PP) during an imagined music performance with that in the resting condition in order to extract connections whose connectivities significantly differed between the two conditions. The results showed increased connectivity of the auditory cortical areas with many non-auditory areas during the music performance task in comparison with the resting condition. The data were acquired in the loud-noise environment of an MRI scanner, which may have affected the activation of the auditory cortical network and limited the emergence of clear patterns of reconfiguration during the imagined music performance task. However, the effect of noise was minimized by subtracting the activity at rest from that observed during the task because the data for both conditions were acquired under the influence of the same noise level. This study shows that the functional network of the auditory cortical areas was dynamically reconfigured during the imagined music performance. In the following sections, we discuss the implications of these results.

### Hemispheric Asymmetry in Functional Connectivity

The enhanced functional network of the auditory cortical areas clearly showed rightward lateralization. This result is consistent with previous results showing that conceiving a mental imagery of music activated auditory cortical areas in the right hemisphere more than those in the left hemisphere (Zatorre and Halpern, 2005; Regev et al., 2021). A voxel-based morphometric study showed increased gray matter concentrations in the right HG in musicians (Bermudez et al., 2009). Acoustic temporal processing has been reported to be weighted toward the left hemisphere, while acoustic frequency processing is weighted toward the right (Zatorre and Belin, 2001). This notion could, at least in part, account for the rightward lateralization of the auditory cortical network identified in this study. However, the extensive connectivity of the auditory cortical network with many associative cortical areas might be associated with a feature that is not restricted to acoustic signal processing. The lateralized auditory network seems to have a structural background: a recent analysis of diffusion imaging data sets showed higher diffusive or integrative intra- and inter-hemispheric connections of the auditory cortex in the right hemisphere than in the left hemisphere (Mišić et al., 2018). The authors argued that “the right auditory cortex is better integrated in the connectome, facilitating more efficient communication with other areas” (Mišić et al., 2018). Taken together, these findings indicate that the auditory cortical network in the right hemisphere is likely to preferentially mediate the integrated mental imagery processing involved in music performance.

### Functional Implications

In a recent psychophysiological interaction analysis of fMRI data acquired from non-musical participants while they listened to music, the researchers argued that HG, PT, and PP all had emotion-characteristic functional connectivity with the limbic/paralimbic structures as well as the visual, somatosensory, and motor areas (Koelsch et al., 2018). There are several hypotheses regarding emotion processing. One of these, the right-hemisphere hypothesis, postulates that emotion is processed predominantly in the right hemisphere (Rohr et al., 2013; Gainotti, 2018). Emotional prosodic processing is also preferentially right-lateralized (Kotz et al., 2006; Sammler et al., 2015). Another hypothesis, the valence hypothesis, suggests that both hemispheres are involved in emotion processing (Rohr et al., 2013). The original valence hypothesis accounts for lateralization of the prefrontal cortex by proposing that positive emotion is processed preferentially in the left hemisphere, while negative emotion is processed preferentially in the right hemisphere. However, the premotor cortex and right temporo-occipital junction seem to be involved in positive emotion processing, while the right temporo-parietal junction seems to be involved in negative emotion processing (Beraha et al., 2012). These results are consistent with the region-specific lateralization hypothesis (Beraha et al., 2012; Zhang et al., 2015). In our study, however, the target regions in the enhanced network extracted do not necessarily coincide with typical emotion-related regions such as the orbitofrontal cortex, insula, and amygdala. Therefore, it seems unlikely that rightward lateralization of the auditory cortical network during imagined music performance is specifically related to emotion processing.

### Network Properties

Our analysis showed different patterns of enhanced connectivity of the three auditory cortical areas considered (HG, PT, and PP) during the imagined music performance task. The detected regions were distributed over the frontal, parietal, temporal, and occipital cortical areas. The seed ROIs in the right hemisphere had more connections with significantly increased connectivity. Overall, the right HG showed enhanced connections with frontal cortical areas more than with other cortical areas; the right PT showed enhanced connections with the frontal, parietal, and temporal cortical areas; and the right PP showed enhanced connections with the temporal, parietal, and occipital cortical areas. Frontal regions, such as the FP, MFG, and SFG, are associated with the mediation of temporal control (Nee and D’Esposito, 2016) and metacognitive control (Fleming and Dolan, 2012). Therefore, it is likely that the frontal regions exerted control over the auditory cortical areas to accomplish the imagined music performance task. The PT and PP, which are higher auditory processing areas, showed increased connectivity with the precuneus during imagined music performance. The precuneus, a principal node of the default mode network (DMN), mediates mental imagery. Enhanced interaction with the precuneus suggests the involvement of these areas in the integration of auditory information with mental imagery for music performance. The generation of mental imagery was supported by the prefrontal cortex and precuneus (Gardini et al., 2009). Therefore, the connectivity of the auditory cortical areas with the prefrontal regions and precuneus in this study suggests that the auditory cortical areas are involved in the generation of musical imagery by interacting with the prefrontal cortex. In addition to cortico-cortical connectivity, all seed regions showed increased connectivity with the thalamus during imagined music performance. This result suggests that cortico-subcortical connectivity also contributes to the process underlying imagined music performance. An fMRI study suggested that the resting-state thalamocortical network between the thalamus and the precuneus is enhanced in musicians than in non-musicians (Tanaka and Kirino, 2017a). Since cortico-thalamocortical networks mediate communication across cortical areas (Sherman, 2012; Bell and Shine, 2016), our results suggest that the auditory cortical areas extend to a large-scale network mediating higher-order integration of sounds and mental imagery.

### Roles in Imagined Performance

Our results suggest that the auditory cortical areas contribute to imagined music performance. To perform this task, the imagination of music performance is required, and the contents of the imagination would include information processing of performance and imagery. Regarding the information processing of performance, we previously analyzed the functional connectivity of the supplementary motor area (SMA) during imagined music performance (Tanaka and Kirino, 2017b). The results showed an increase in its connectivity with the dorsolateral prefrontal, sensorimotor, parietal, posterior temporal, and occipital cortices during imagined music performance, suggesting the SMA’s involvement in performance planning. The target ROIs with significant increases in connectivity with the auditory cortical areas did not include the SMA in this study, suggesting that the auditory cortical areas do not contribute to performance planning. In contrast, HG and the PT increased their connectivity with the AG, a principal node of the DMN. Our previous study showed that imagined music performance increased the functional connectivity of the AG with the other nodes of the DMN, which processes mental imagery (Tanaka and Kirino, 2019). Taken together, these results suggest that the auditory cortical networks contribute to the processing of musical imagery rather than performance planning.

### Limitations

Musical imagery is multi-faceted (Cotter, 2019). This study did not assess whether musical imagery is broken down into general imagination processes and imagery specific to music. Further, since all participants are well-trained classical music students, as described in section “Participants,” their musical imagery is likely to be coupled with the imagery of performance. The processing of performance imagery might also include the generation of imagery. For the same reason, the results of this study, especially the right-lateralized enhancement of functional connectivity of the auditory cortical areas, might be restricted to such experts. It would be interesting to see the results in non-musicians. However, the imagined music performance might be unaccomplishable unless one has an experience of active participation in concert performances. Therefore, it would be reasonable to state that the results of this study is applicable only to music experts. All participants in this study are female. Although the results would be unlikely to differ significantly, this study did not assess gender effects.

We instructed the participants to remain still during the scans and checked by sight. After each session, we asked them if they had made no movements and confirmed it. We did not monitor electromyography. Regarding imagery, we admit that such subjective processing is difficult to control, although subjectivity was inherently important for this study. In the future, a better method needs to be developed to overcome it.

## Conclusion

The extracted auditory cortical network during imagined music performance relative to the resting condition showed enhanced functional connectivity of the auditory cortical areas with many higher-order cortical areas. The network reconfiguration showed a clear rightward lateralization and included target regions in the prefrontal, temporal, and cortical midline structures, suggesting highly associative processing. Herein, we propose that auditory cortical areas, and their networks are actively involved in imagined music performance through the integration of auditory imagery into mental imagery associated with the performance.

## Data Availability Statement

The original contributions presented in the study are included in the article, further inquiries can be directed to the corresponding author.

## Ethics Statement

The studies involving human participants were reviewed and approved by the Ethics Committees of Sophia University and Juntendo University, Japan. The patients/participants provided their written informed consent to participate in this study.

## Author Contributions

ST and EK planned and conducted all the experiments. ST analyzed the data and wrote the manuscript. Both authors contributed to the article and approved the submitted version.

## Conflict of Interest

The authors declare that the research was conducted in the absence of any commercial or financial relationships that could be construed as a potential conflict of interest.

## Publisher’s Note

All claims expressed in this article are solely those of the authors and do not necessarily represent those of their affiliated organizations, or those of the publisher, the editors and the reviewers. Any product that may be evaluated in this article, or claim that may be made by its manufacturer, is not guaranteed or endorsed by the publisher.

## Footnotes

1
http://www.fil.ion.ucl.ac.uk/spm
